# A study of laser surface treatment in bonded repair of composite aircraft structures

**DOI:** 10.1098/rsos.171272

**Published:** 2018-03-21

**Authors:** Shaolong Li, Ting Sun, Chang Liu, Wenfeng Yang, Qingru Tang

**Affiliations:** Aviation Engineering Institute, Civil Aviation Flight University of China, Guanghan 618307, People's Republic of China

**Keywords:** laser surface treatment, interface, bonded repair, carbon fibre reinforced polymer

## Abstract

Surface pre-treatment is one of the key processes in bonded repair of aircraft carbon fibre reinforced polymer composites. This paper investigates the surface modification of physical and chemical properties by laser ablation and conventional polish treatment techniques. Surface morphology analysed by laser scanning confocal microscopy and scanning electron microscopy showed that a laser-treated surface displayed higher roughness than that of a polish-treated specimen. The laser-treated laminate exhibited more functional groups in the form of O 1 s/C 1 s atomic ratio of 30.89% for laser-treated and 20.14% for polish-treated as evidenced by X-ray photoelectron spectroscopy observation. Contact angle goniometry demonstrated that laser treatment can provide increased surface free energy and wettability. In the light of mechanical interlocking, molecular bonding and thermodynamics theories on adhesion, laser etching process displayed enhanced bonding performance relative to the polishing surface treatment. These properties resulted in an increased single lap shear strength and a cohesive failure mode for laser etching while an adhesive failure mode occurred in polish-treated specimen.

## 1. Introduction

In the last decade, carbon fibre reinforced polymer (CFRP) has been successfully used in civil aircraft primary structural applications (e.g. nearly 53 wt% composite materials of Airbus 350 and 50 wt% of Boeing 787) [1,2]. Compared with metals, advanced composite materials can provide superior properties such as light weight and high rigidity, corrosion and fatigue resistance, low fuel consumption and emission reduction. Likewise, a composite fuselage can provide comfortable humidity and cabin pressure for passengers. Excellent tailored properties and improved maintenance check period make the composite aircraft gaining in popularity [3,4]. However, the big differences of anisotropic and heterogeneous behaviours make it much more complex from the aspect of non-destructive test for damage assessment, maintenance theories, process and methods, repair verification and evaluation, continued airworthiness and safety test than traditional metal construction [5]. The National Aeronautics and Space Administration clearly emphasized that the major challenges for the composites aircraft industry are CFRP structural maintenance and repair technologies [6,7]. Metal structural repair often uses riveting and screw connecting techniques while composite repair can make use of bonded repair (sided lap or scarf based) to provide an aerodynamically smooth surface [8–10].

The initial CFRP material surface is inappropriate for bonded repairs due to some disadvantages such as surface contamination, irregular appearance, bad wettability, low surface free energy and chemical inertness. Therefore, surface pre-treatment is a very important processing step before adhesive bonding of the substrate materials to improve the adhesion behaviour and enhance the maintenance quality and reliability [11–21]. Manual polishing and mechanical abrasion are the common surface treatments of bonded composite repair. Other pre-treatment processes like grit blasting, corona discharge, plasma modification, peel-ply and chemical etching techniques have also gained extensive attention of academia and industry [22–36]. However, these methods exhibit various shortcomings. Mechanical abrasion is an inefficient long-period process and unable to avoid human errors and inconsistencies. Corona discharge and plasma treatment could activate the surface molecules to modify the chemical property and wettability while the equipment is expensive and the depth of processing is only a very thin layer. Peel-ply is short-term storage and needs cryopreservation. With regard to chemical etching method, solutions like strong acid/alkali/oxidant will pollute the environment and are harmful to operators. Besides, it can also damage the matrix or reinforced fibres.

Recently, laser surface treatment of composite materials has attracted attention, as a kind of new and high-functionality technique. Conventional surface preparation usually damages the substrate leading to reinforced fibre breaking or peeling. Furthermore, it is easy to produce secondary pollution for these processing methods. On the contrary, laser surface preparation is a non-contact and green process, which can be easily controlled and automated. In addition, the strong and accurate laser beam energy can change both the chemical and physical behaviour of the surface through laser etching process by adjusting the laser parameters (e.g. working nominal power, wavelength, pulse width). Laser-treatment process can provide uniform surface behaviours to ensure high reliability and repeatability for aircraft structural repairs to avoid human errors and inconsistencies. Reitz demonstrated that wavelength and energy input are the critical parameters for laser pre-treatment, and UV-laser system can avoid near surface laminate damage while IR-laser breaks the fibre--matrix [37]. Henrik used a Nd:YAG laser to study the influence of adhesive properties in a standard SCARF repair process and showed that oxidized fibres can improve the adhesive strength [38]. Palmieri found that a 355 nm Nd:YAG laser could ablate about 10–20 µm depth of the laminate surface without breaking fibres and will improve the reproducibility and robustness in a production environment [39]. Belcher proved that higher surface energies could result in enhanced shear bond strengths while laser etching and peel ply treatment displayed equal adhesion values, better than grit blasting process [40]. Volkermeyer presented an IR-laser ablation with increased wettability and surface energy in relation to surface grinding specimens [41].

Although the fundamental mechanisms for adhesion are not yet completely understood, some theories such as mechanical interlocking, chemical bonding and thermodynamics theories on adhesion have been proposed [42–46]. Parameters like surface morphology, roughness, active functional group, surface free energy and wettability can greatly influence the adhesion properties. In this paper, a systematic investigation based on laser-treated and polish-treated samples will assist in determining the various factors in different theories. Furthermore, single lap shear strength tests and failure mode definitions according to ASTM D5573-99 (2005) were examined to verify these theories.

## 2. Material and methods

### 2.1. Materials

The investigated composite panel was 2 mm thick Boeing carbon fibre reinforced plastic (T300 CFRP/epoxy resin, PWC T300/YCOM970) laminate with 16 plies of a [0°, ±45°, 0°]_2_ satin weave prepreg. The parent laminate was fabricated in an autoclave with a heating rate of 3°C min^−1^ to 180°C and then stayed for 2 h. Subsequently, it was cooled to room temperature with the same rate. The obtained laminate was cut into small square plates (5 cm × 5 cm) for further preparation.

### 2.2. Laser and polish activation of the bonding surfaces

We chose polish-based treatment of the panel to compare with laser-based treatment of the bonding surface due to the dramatic differences of the preparation process and the interface behaviours after activation. The laser pretreatment patch was an automated process. We just tore off the peel ply from the laminate to show up the top-layer epoxy resin, then put the samples on the operations area to activate the surface by adjusting the laser parameters. Laser ablation of CFRP parent laminates was accomplished through Nd: YAG laser equipment of Inno Laser Corp. The working nominal power of the system was equal to 1.4 W (10% laser power) output at 1064 nm and a 30 ns pulse width. The other parameters were 100 kHz frequency, 1000 mm s^−1^ scan speed, 50 µm beam spacing and 50 µm beam width with two passes. The pulse energy at the ablation area was calculated as about 700 mJ cm^−2^. We chose these main laser parameters based on the damage threshold of the polymer matrix and the fibre (i.e. beyond the polymer's ablation threshold and below the fibre's ablation threshold), which means to remove the epoxy resin without breaking the carbon fibre.

Another surface preparation was mechanical polishing abrasion. The abrasive process was a multilevel polishing treatment (wet sanding on an automated polishing machine (MP-1B, Wanheng, Shanghai, China) with 500 up to 1500 grain size sanding paper and polishing cloth) to achieve exceptional smooth effect. The rotating speed and process time were chosen as 1000 r.p.m. and 2 min per period. Then we used acetone solvent to clean the contaminants and debris in an ultrasonic cleaner, finally drying the surface of laminates.

### 2.3. Laser scanning confocal microscopy

Surface structures and roughness of the target laminates were observed using a laser scanning confocal microscope (LSCM; VK-9710, Keyence, Japan). The chosen scanning wavelength of the microscope was 408 nm. The classical parameter *Ra* (equation (2.1), where *Z* is the altitude difference of a given point having *x* coordinate) is based on the linear contour method, while the characteristic parameter *Sa* (equation (2.2), where *Z* is the altitude difference of a given point having *x*,*y* coordinates) is based on the region contour method. *Sa* is more suitable for surface representation of micro-profile and anisotropic composite materials. Five measurements of each sample were gathered to achieve average values of the roughness *Sa*.

2.1 $$Ra=\left(\frac{1}{N}\right)\sum_{x=1}^{N}\left|Z_{x}\right|$$

and

2.2 $$Sa=\left(\frac{1}{NM}\right)\sum_{x=1}^{N}\sum_{y=1}^{M}\left|Z_{xy}\right|.$$

### 2.4. Scanning electron microscopy

Scanning electron microscopy (SEM; FEI, Hillsboro, USA) was performed with an accelerating voltage of 5 kV to observe the modified morphology of the surface treatment samples. The specimens did not need to be sputtered with a gold coating prior to measurement because of the good electrical conductivity of CFRP and the new technologies used in this instrument, thereby avoiding the disturbance of the gold layer.

### 2.5. X-ray photoelectron spectroscopy

X-ray photoelectron spectroscopy (XPS) analysis was used to investigate the chemical composition of the surface through Axis Ultra DLD apparatus (Kratos Analytical, Manchester, UK). Monochromated AlK*α* radiation (*hv *= 1486.6 eV) was used for bombardment of the surface to elicit the emission of photoelectrons with element-specific binding energy (BE). Each sample was observed at the energy range of 0–1350 eV. The measured BE peak position and areas determined the elements and groups according to Shirley background subtraction.

### 2.6. Contact angle goniometry

The contact angles for the surfaces of laser-based treatment and polish-based treatment were tested with a contact angle analyser (First Ten Angstroms 1000B system, UK). The laminates were flat on the sample platform, then about 3 µl probe purified water and glycerin were introduced on the surface of the panels, respectively, with the sessile drop technique. The software will automatically photograph and determine the contact angles after 30 s until the drops on the panel are unchanged. At least five measurements were collected for each sample to obtain the average result.

### 2.7. Single lap shear tests

**Figure 1.**
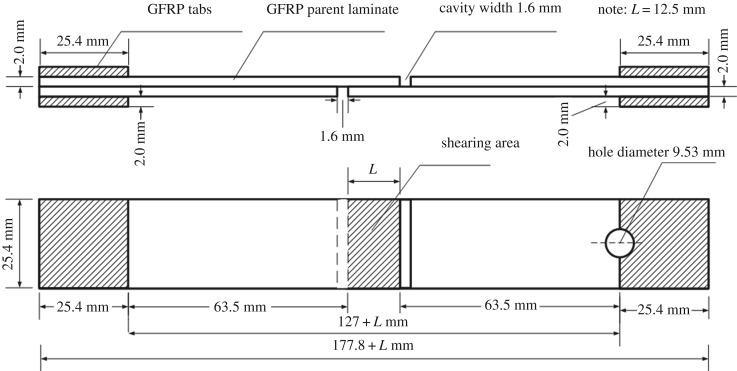
Scheme of single lap shear test samples configuration according to ASTM D3165-07 (2014) and as modified in present work (drawing not to scale).

Single lap shear tests were chosen to characterize the adhesive property after different surface activation. The fabrication method of specimens was according to ASTM D3165-07 (2014) standard with a slight modification. For mechanical tests, GFRP tabs (25.4 mm × 25.4 mm area, 2 mm thick) were placed on the bonded samples to ensure enough clamp force and avoid specimen slippage. Two panels were step bonded with a thickness of 0.125 mm using epoxy adhesive (BMS5-154, 3 M, St Paul, Minnesota, USA, usually used in the composite repair) only in a 12.5 mm × 25.4 mm area were pretreated by laser or polishing. The curing process used vacuum bagging instead of autoclave to simulate the normal aircraft composite bonded repair, and the experimental temperature did not change. Shear strengths of samples were measured with an Instron testing machine (model 8801, Instron Engineering Corporation, Canton, MA) at a displacement rate of 1 mm m^−1^ at ambient temperature. In the lap shear test, we measured the real overlap dimension (width × length) and used it to calculate the shear strength. A minimum of five measurements of each sample were gathered to achieve average values of shear stress. The specimen dimensions can be seen in figure 1.

## 3. Results and discussion

### 3.1. Surface morphology

The surface geometric construction of adherend is one of the key parameters for bonded repair according to the mechanical adhesion theory raised by Bcbain [43]. Modest increase in surface roughness would enhance the ‘mechanical interlocking’ among the adherend and adhesive. The target panels (5 cm × 5 cm) were prepared via laser-treatment and polish-treatment, respectively, to achieve various geometrical morphologies of activated bond surface. The details of the manufacturing processes are in §2.2.

**Figure 2.**
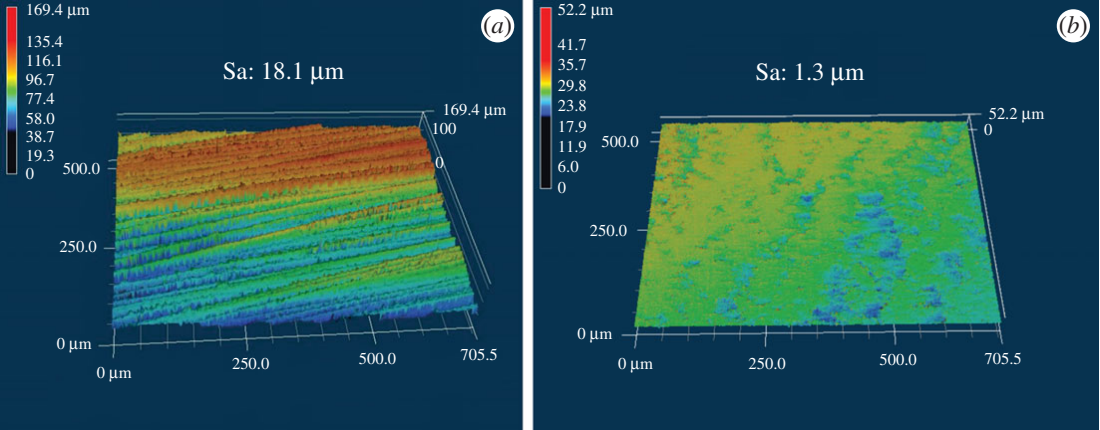
LSCM images of CFRP composite surfaces: (*a*) laser-treated, Sa = 18.1 µm; (*b*) polish-treated, Sa = 1.3 µm.

LSCM images of CFRP laminates with two different methods are shown in figure 2 which indicate that the laser etching process produced deeper trenches while the polish preparation resulted in a smooth surface. The roughness *Sa* calculated by surface profilometry was 18.1 ± 1.1 µm for laser-treated sample, 1.3 ± 0.3 µm for polish-treated sample. Moreover, the surface roughness of the untreated sample was nearly 0.6–1.7 µm (based on the dust and any other contaminant above it, not shown here) which means laser treatment greatly increased the surface roughness.

**Figure 3.**
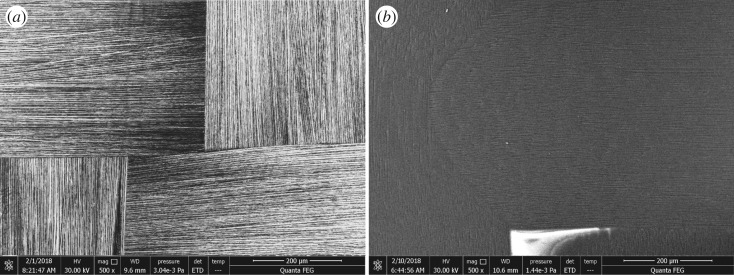
SEM images (scale bar = 200 µm) of CFRP composite surfaces: (*a*) laser-treated; (*b*) polish-treated.

For further study of the surface feature of the activated area, laminates were measured by SEM. Figure 3 shows SEM micrograph of each activated panel. The polymer matrix was partially wiped in the polished samples while fibres were found intact (figure 3*b*). This arises from the soft property of the polishing cloth that can only wipe off epoxy resin except for hard carbon fibres under the polymer layer. If, on the other hand, the laser is used, then removal level can be controlled precisely via tuning the parameters. During the ablation period, the physical and chemical properties of CFRP surface can be both modified. Figure 3*a* shows the activated surface of laser-treated sample, where most of the resin was removed and the fibre bundles appeared without degradation. The contaminant and debris generated from the laser etching were also blasted off due to thermal energy, and this work would not need additional operations (e.g. polish/solvent wiping). It should be noted that the laser parameters we chose were according to below the ablation threshold of the fibre reinforcement to avoid damage of the fibres [47]. The influences of various laser parameters (e.g. laser type, laser wavelength, pulse width, scan speed, laser power) were not considered and would be researched in our future study.

### 3.2. Surface chemical composition

**Figure 4.**
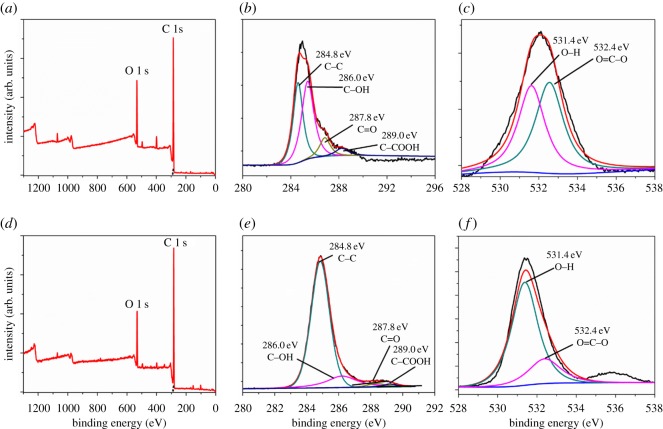
XPS curves of CFRP composite surfaces: (*a*) C 1 s and O 1 s peaks were analysed for laser-treated sample; (*b*) deconvolution bands of the XPS C 1 s binding energy for laser-treated sample; (*c*) deconvolution bands of the XPS O 1 s binding energy for laser-treated sample; (*d*) C 1 s and O 1 s peaks were analysed for polish-treated sample; (*e*) deconvolution bands of the XPS C 1 s binding energy for polish-treated sample; (*f*) deconvolution bands of the XPS C 1 s binding energy for polish-treated sample.

Based on the chemical adhesion theory, increase in the chemical bonding activity between the adherend and the adhesive layer can obviously enhance the joint strength [46]. Laser treatment was used to selectively remove the laminate surface by laser ablation. Physico-chemical reaction occurred simultaneously which revealed an increase of surface roughness and oxidation degree. XPS analysis is an effective approach to study the modification of surface elements and chemical bonds, and the results are presented in figure 4 and table 1. Each activated surface shows O 1 s peak and C 1 s peak that indicate that both samples contain oxygen group. The O 1 s/C 1 s atomic ratio of polish-treated sample was 20.1%, and that of the laser-treated sample was 30.9%. It means the surface of laser-treated panel presents more functional groups in favour of composite bonded repair. Figure 4*b,e* shows the deconvolution bands of the XPS C 1 s peak. The spectra show four characteristic peaks for each sample and the BE peaks located at 284.8, 286.0, 287.8 and 289.0 eV were corresponding to C–C, C–OH, C–COOH and C=O groups accordingly. Analogously, the changes of oxidation state of O 1 s peak are deconvoluted in figure 4*c* and figure 4*f*, respectively. The BE peaks located at 531.4 and 532.4 eV were a response to O–H and O=C–O accordingly. To further investigate the percentage content of surface chemical composition with different treatment, peak areas at a given position were integrated and the values are given in table 1. The data showed that the content of low active C–C group of laser-treated sample was less than polish-treated one; on the contrary, laser-treated sample exhibited more functional groups (i.e. the other three groups). The content of BE peaks located at 531.4 (O–H) for polish-treated sample was more than 77% which was produced by the curing of epoxy resin. After laser ablation, the single-bonded oxygen (O–H) was further oxidized to double-bonded oxygen (O=C–O). It resulted in higher O=C–O content and lower O–H content for laser-treated specimen. Chemical analysis identified that laser etching produced higher activity compared with polish preparation.

**Table 1.** XPS data of activated surface.

sample	C 1 s^a^	O 1 s^a^	O 1 s/ C 1 s ratio (%)	C–C^b^	C–OH^b^	C–COOH^b^	C=O^b^	O–H^c^	O=C–O^c^
laser-treated	70.2	21.7	30.9	38.8	47.3	4.3	9.6	49.6	50.4
polish-treated	78.5	15.8	20.1	69.7	4.0	1.8	3.1	77.2	22.8

^a^Atomic percentage (%).

^b^Deconvolution bands of the XPS C 1 s peak.

^c^Deconvolution bands of the XPS O 1 s peak.

### 3.3. Surface free energy

Another theory describing thermodynamic mechanism was investigated using wettability (a general parameter) to further research the surface features of various treatment methods of CFRP. It is believed that the interface bond strength is mostly derived from the molecular adsorption interactions between the adhesive and adherend. This theory is based on the surface free energy that combines polar and dispersive components. High surface energy shows good wettability and strong interface adhesion in bonded repair of composites. The surface energy could be calculated by modification of the Laplace–Young method and Owens–Wendt–Rabel–Kaelble (OWRK) geometric mean theory as follows:

3.1 $$\gamma_{\mathrm{LG}}(1+\cos\theta )=2\sqrt{\gamma_{\mathrm{SG}}^{d}\gamma_{\mathrm{LG}}^{d}}+2\sqrt{\gamma_{\mathrm{SG}}^{p}\gamma_{\mathrm{LG}}^{p}},$$

where *θ* is the measured contact angle of each liquid; $\gamma_{\mathrm{LG}}^{}$, $\gamma_{\mathrm{LG}}^{p}$ and $\gamma_{\mathrm{LG}}^{d}$ are the liquid's surface tension, surface energy of polar component and dispersive component, respectively (table 2). $\gamma_{\mathrm{SG}}^{p}$ and $\gamma_{\mathrm{SG}}^{d}$ are the solid’ surface energy of polar component and dispersive component, respectively. In addition, the total surface energy $\left(\gamma_{\mathrm{SG}}^{}\right)$ of activated materials is the sum of the polar and dispersive components.

**Table 2.** The characteristic parameters of both liquids and various composite surface energies (mJ m^−2^).

liquid	$\gamma_{\mathrm{LG}}$	$\gamma_{\mathrm{LG}}^{d}$	$\gamma_{\mathrm{LG}}^{p}$	sample	$\gamma_{\mathrm{SG}}^{}$	$\gamma_{\mathrm{SG}}^{d}$	$\gamma_{\mathrm{SG}}^{p}$
water	72.8	21.8	51	laser-treated	62.5	47.4	15.1
glycerin	64	34	30	polish-treated	48.8	45.8	3.0

**Figure 5.**
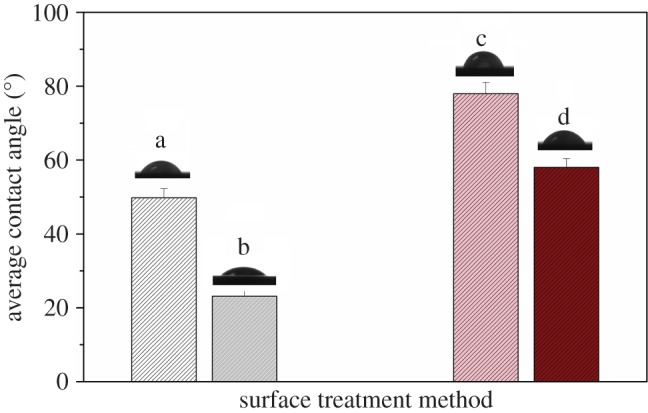
Contact angle of samples with various preparation conditions. a, Water contact angle for laser-treated sample; b, glycerin contact angle for laser-treated sample; c, water contact angle for polish-treated sample; d, glycerin contact angle for polish-treated sample.

Four average contact angles of various liquids and surface preparation conditions are shown in figure 5. Laser-treated sample displayed a lower contact angle for each liquid which means the surface exhibits better wetting behaviour. In addition, we calculated the surface free energy of different pre-treated panels due to OWRK theory. Table 2 shows that both preparation techniques yield comparable disperse part of surface energy while laser-treated one yields a markedly higher polar part of surface energy. The total surface energy of laser and polish pre-treatment is 62.5 and 48.8 mJ m^−2^, respectively. Our previous research showed that the surface free energy value for untreated sample was about 43.5 mJ m^−2^ [48]. Compared with the polish-treated surface, the higher determined free energy for the laser-ablated surface confirmed the XPS data suggesting the presence of more high surface energy chemical species on the laser-treated surface. Laser-activated technique resulted in significant increase of the surface energy indicating the adhesive could completely infiltrate the adherend to provide a considerable bond strength and improve the joint efficiency.

### 3.4. Mechanical testing and failure mode

**Figure 6.**
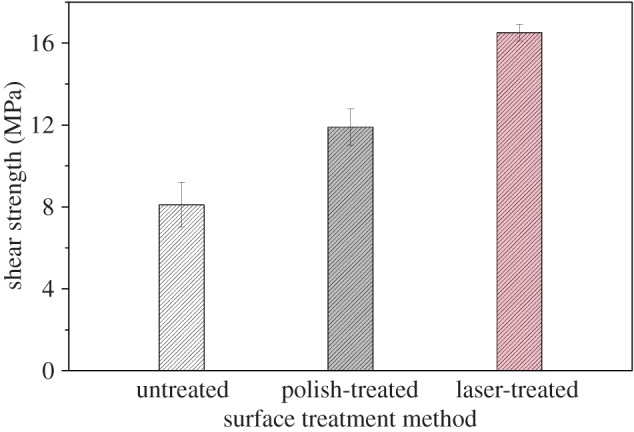
Single lap shear results of samples with various preparation conditions (untreated, polish-treated and laser-treated specimens).

Apparent shear strength tests have been conducted on CFRP samples as described in §2.7. In order to illuminate the influence of different treatments on the laminate surfaces, initial material named untreated sample was used for comparison. As can be seen from figure 6, the lap shear strength of untreated adherend was determined to be 8.1 ± 1.1 MPa, while the shear bonding strength averaged 11.9 ± 0.9 MPa for polish-treated sample and 16.5 ± 0.4 MPa for laser-treated one. According to the analysis of mechanical adhesion theory, chemical bonding activity and thermodynamic mechanism, laminates with rough surface, activated chemical composition and surface free energy could meet satisfactory maintenance effect which show higher mechanical strength. The untreated specimen showed a poor adhesion due to the dust and any other surface contaminants. Laser-treated specimen provided sharply enhanced mechanical properties compared with polish-treated one for its increased roughness, chemical modification and wettability. Moreover, the value of standard deviation of laser-etched laminate is the least of the three samples. The experimental results showed that laser surface procedure can provide uniform and consistent surface properties in comparison to conventional mechanical abrasion surface preparation. The essential reason was that manual repair cannot make every abrasion keep the same conditions (e.g. time, force direction and pressure, friction cycle times). However, the laser treatment was an automated process which could achieve robust, reliable and repeatable bonded repairs.

**Figure 7.**
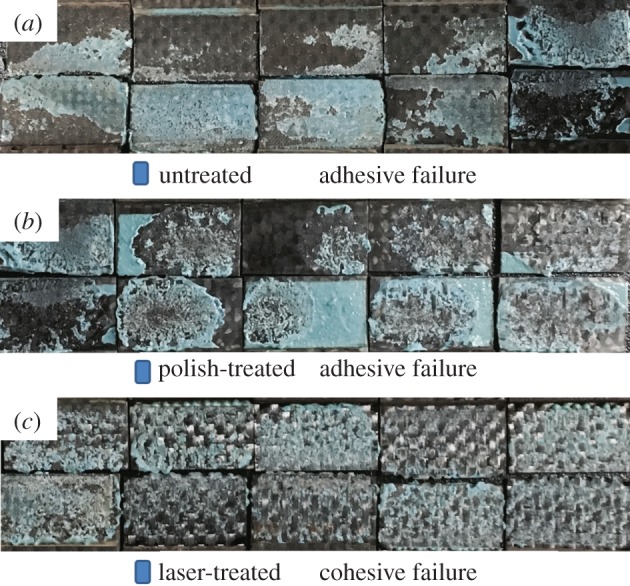
Failure mode of samples with various preparation conditions (untreated, polish-treated and laser-treated specimens).

The failure mode definitions of the three chosen specimens were according to ASTM D5573-99 (2005). The untreated samples had an adhesive failure mode while laser-ablated samples had a cohesive failure mode. The polish-treated samples resulted in major adhesive and slightly cohesive failure modes which were assigned to adhesive failure mode (figure 7). The fracture patterns revealed that the laser treatment technique produced high performance adhesion which is observed as completely cohesive failure indicating a sharply increased adhesive bonding strength compared to the conventional techniques.

## 4. Conclusion

In this research, two different activated composite laminates were manufactured through polish and laser treatment to study the surface behaviours and adhesion properties in bonded repair of CFRP aircraft structures. Three different adhesion theories (i.e. mechanical interlocking, chemical bonding and thermodynamics) were employed to reveal the surface bonding mechanism. The results demonstrate that laser-treated sample displays greater roughness, active functional groups and wettability than polish-treated specimen. Finally, single lap shear strength was tested according to ASTM D3165-07. Laser etching showed good adhesive strength and cohesive failure mode. On the contrary, polish treatment technique showed poor bonding force and adhesive failure mode. Laser surface treatment is a green and non-touch procedure without using solutions or introducing secondary contaminations. Compared with conventional mechanical abrasion, laser-treated process can provide uniform surface behaviours to ensure high reliability and repeatability for aircraft structural repairs to avoid human errors and inconsistencies.

Further investigations will be focused on the influence of laser parameters (e.g. working nominal power, wavelength, pulse width) on the modification of CFRP physical and chemical properties of the surface. Then, optimized laser process will be compared with distinct mechanical grinding (e.g. grid 60 120 240 360 and polish) to study the industrial application of laser surface treatment techniques.

## Data accessibility

All the performance data are provided in the main text. There are no additional data associated with this submission.
